# Genome Sequence of *Arthrobacter antarcticus* Strain W2, Isolated from a Slaughterhouse

**DOI:** 10.1128/genomeA.00073-16

**Published:** 2016-03-31

**Authors:** Jakob Herschend, Prem K. Raghupathi, Henriette L. Røder, Søren J. Sørensen, Mette Burmølle

**Affiliations:** Section for Microbiology, Department of Biology, University of Copenhagen, Copenhagen, Denmark

## Abstract

We report the draft genome sequence of *Arthrobacter antarcticus* strain W2, which was isolated from a wall of a small slaughterhouse in Denmark. The 4.43-Mb genome sequence was assembled into 170 contigs.

## GENOME ANNOUNCEMENT

*Arthrobacter antarcticus* is a Gram-positive, aerobic, motile bacterium having a rod-coccus cycle, i.e., fragmentation of the rods and filaments forms the next generation of cocci or short rods (1). *A. antarcticus* was reported to have been originally isolated from Antarctic marine sediments (2). The genus *Arthrobacter* contains bacterial species of high diversity, which are often found in soil (3), alpine ice caves (4), wastewater reservoir sediment (5), cheese surfaces (6), clinical specimens (7), and fish (8). Here, we present the draft genome sequence of *Arthrobacter antarcticus* strain W2 isolated from a wall in a slaughterhouse in Denmark. In a recent study, we have shown that *Arthrobacter antarcticus* strain W2 can interact with other bacterial species isolated from the same environment to form synergistic biofilms (9).

The sequence library of *Arthrobacter antarcticus* W2 was prepared using the Nextera XT kit (Illumina, USA), according to the manufacturer’s recommendations, followed by sequencing as a part of the flowcell, as 2 × 250-base paired-end reads, using Illumina MiSeq (Illumina, USA) technology. The resulting reads were trimmed and merged with CLC Genomics Workbench 7 (CLC Bio-Qiagen, Aarhus, Denmark). Both merged and unmerged reads were assembled in SPAdes 3.5.0 (10). Contigs were screened against the VecScreen database to ensure that contigs were without Nextera sequence contamination. Contigs smaller than 500 bp were discarded. Final contigs were annotated using the RAST server (11) and RNAmmer 1.2 (12) to screen for noncoding rRNAs and tRNAs. The draft genome of *A. antarcticus* W2 was 4,443,083 bp long, with coverage of 50×, and was assembled into 170 contigs with an average G+C content of 61.7% and 81 RNA genes. RNAmmer analysis predicted 4 copies of 5s and 1 copy each of 23s and 16s rRNA genes.

The annotated genome sequence revealed a total of 4,038 coding regions out of which 1,534 were functionally annotated. The coding sequence of this strain contained genes for capsular and extracellular polysaccharides, metabolism of aromatic compounds, and stress responses. Genes for virulence, disease, and defense, including genes for resistance against vancomycin, beta-lactamase, fluoroquinolones, arsenic, and mercury, were identified. Genes for heavy metal resistance, like copper tolerance and cobalt-zinc-cadmium resistance, were also identified. In addition, this species possessed genes facilitating invasion and intracellular resistance. Functional comparison available on the RAST server revealed *Arthrobacter* sp. FB24 (score: 507) followed by *Arthrobacter chlorophenolicus* A6 (score: 499) to be the closest neighbors of *A. antarcticus* W2. Certain features mentioned above can have medical and industrial implications, and the availability of this draft genome can promote future functional and comparative analyses of the genome.

### Nucleotide sequence accession numbers.

The whole-genome shotgun project for *A. antarcticus* W2 has been deposited in the European Nucleotide Archive (ENA) under the contig accession numbers CZJT01000001 to CZJT01000170. The version described in this paper is the first version.
